# The importance of precise plane selection for female adult Chiari Type I malformation midsagittal morphometrics

**DOI:** 10.1371/journal.pone.0272725

**Published:** 2022-08-10

**Authors:** Mark Morkos, Maggie Eppelheimer, Blaise Simplice Talla Nwotchouang, Seyed Amir Ebrahimzadeh, Rafeeque A. Bhadelia, Dorothy Loth, Philip A. Allen, Francis Loth

**Affiliations:** 1 Conquer Chiari Research Center, Department of Biomedical Engineering, The University of Akron, Akron, OH, United States of America; 2 Department of Radiology, Beth Israel Deaconess Medical Center, Harvard Medical School, United States of America; 3 Conquer Chiari Research Center, Department of Psychology, The University of Akron, Akron, OH, United States of America; 4 Departments of Mechanical and Industrial Engineering and Bioengineering, Northeastern University, Boston, MA, United States of America; Harvard Medical School, UNITED STATES

## Abstract

**Introduction:**

Morphometric assessment of Chiari malformation type I (CMI) is typically performed on a midsagittal MRI. However, errors arising from an imprecise selection of the midsagittal plane are unknown. We define absolute parasagittal error as the absolute difference between morphometric measurements at the midsagittal and parasagittal planes. Our objective was to determine the absolute parasagittal error at various lateral distances for morphometric parameters commonly used in CMI research.

**Methods:**

Sagittal T1-weighted MRI scans of 30 CMI adult female subjects were included. Image sets were evaluated to assess 14 CMI morphometric parameters in the midsagittal plane and four parasagittal planes located 1 and 2 mm lateral (left and right). Comparisons between measurements at the midsagittal and parasagittal planes were conducted to determine the mean individual absolute and mean group parasagittal errors for all 14 parameters.

**Results:**

The mean individual absolute parasagittal error was > 1 unit (1 mm for lengths and 1 degree for angles) for 9/14 parameters within a lateral distance of 2 mm. No significant group parasagittal errors were seen in 14/14 parameters, including tonsillar position within a lateral distance of 2 mm.

**Conclusion:**

Our results show that the absolute errors for imprecise midsagittal plane selection may impact the clinical assessment of an individual patient. However, the impact on group measurements, such as in a research setting, will be minimal.

## Introduction

Chiari malformation type I (CMI) is a neurological condition characterized by cerebellar tonsils that extend below the foramen magnum at the base of the skull. CMI patients experience a range of symptoms, including headaches, retro-orbital pain, ocular disturbances, cough-associated headache, neck pain, cognitive dysfunction, depression, anxiety, balance problems, and numbness [1–5]. The current diagnosis process utilizes imaging of the cerebellar tonsils, patient-reported symptoms, and neurological signs. One area of CMI research involves morphometric measurements evaluated on structural magnetic resonance images (MRI) [2, 4, 6–10]. Eppelheimer et al. illustrated the significance of morphometric measurements to identify prevalent conditions related to CMI morphometrics [8]. They found that McRae’s line and basion to posterior axial line were significantly different in cases with certain prevalent conditions than those without such conditions. In another study, Yucel et al. revealed the use of morphometric measurements in diagnosing Alzheimer’s disease, which further highlights morphometric measurements as a potential diagnostic tool for various neurological conditions [10].

**Fig 1:**
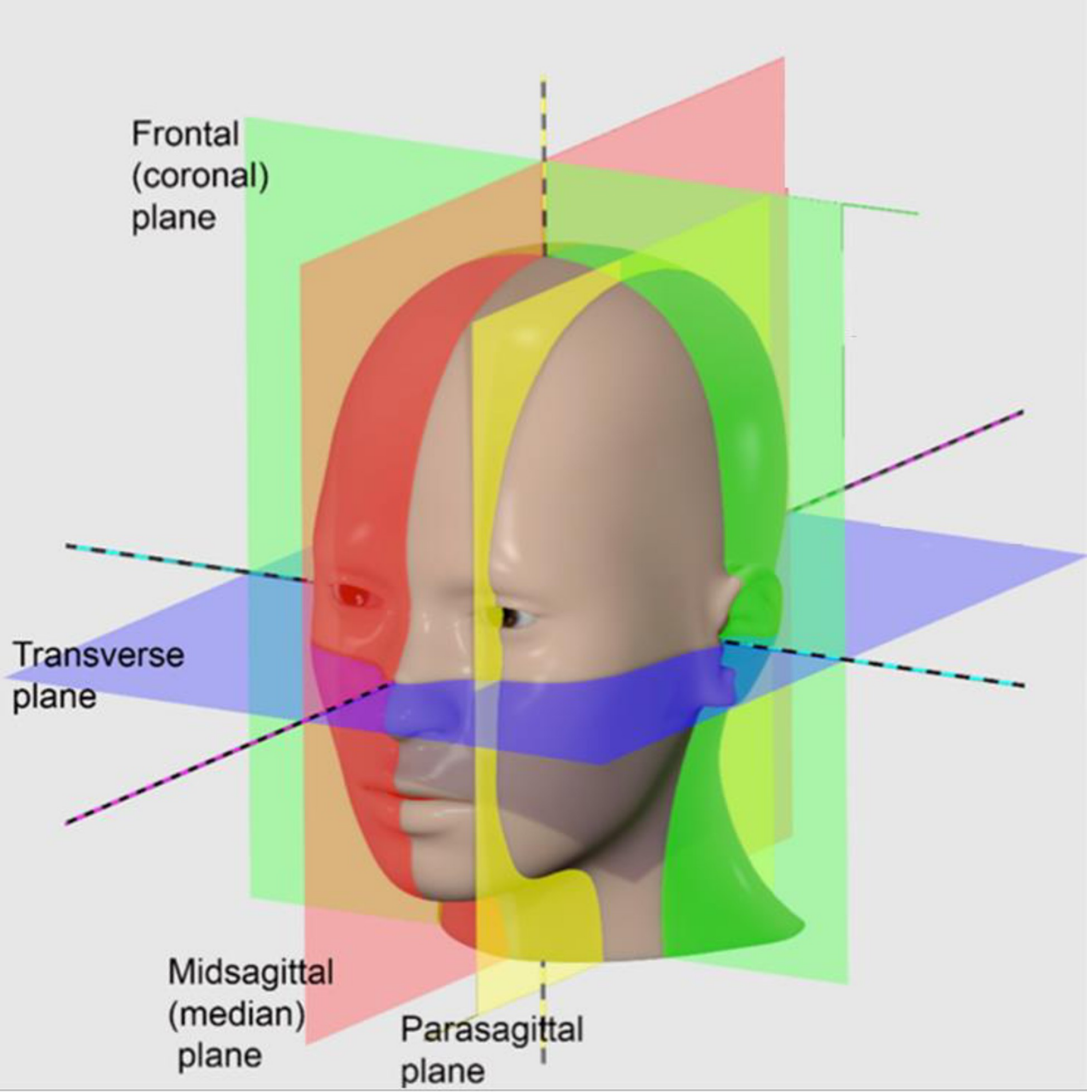
Representation of midsagittal and parasagittal planes. Reproduced from [11] (CC BY-SA 4.0).

Typically, morphometric measurements are obtained from an image of the median plane of the head, known as the midsagittal plane shown in Fig 1 [11]. The midsagittal plane is identified using a set of criteria, specifically visibility, or clear definition of four structures: the genu of the corpus callosum, the splenium of the corpus callosum, the infundibulum, and the cerebral aqueduct [8]. Obtaining the midsagittal plane slice using these criteria allows morphometric measurements from different individuals to be compared and analyzed. Wu et al. used parameterized surface matching to automatically extract the midsagittal plane from a sequence of MRI slices, to allow morphometric measurements to be more easily obtained [12]. In another study, Kuijf et al. developed a technique based on the Kullback-Leibler measure to identify and segment the midsagittal plane [13].

A recent study conducted measurements of tonsillar position in two parasagittal planes on each side of the brain by locating the tonsillar tips on the coronal plane [2]. Ebrahimzadeh et al. assessed diagnostic utility by comparing parasagittal plane measurements to the midsagittal measurements in the prediction of cough-associated headache. They demonstrated that a parasagittal measurement of the smaller tonsillar herniation was a more effective indicator to diagnose cough-associated headaches than midsagittal measurements. The researchers recommended that an analysis of other parameters of the posterior fossa area across parasagittal planes would be beneficial in identifying more effective diagnostic utilities for CMI.

Morphometric measurement errors can arise if the sagittal image is inadvertently selected lateral to the midsagittal plane at a parasagittal plane. No previous studies have examined the difference in morphometric measurements due to imprecise selection of the midsagittal plane. We define parasagittal error as the difference between morphometric measurements at the midsagittal and parasagittal planes. For lengths, the parasagittal error is the numerical difference, in mm, of the morphometric measurement at a particular parasagittal slice and the morphometric measurement at the midsagittal slice. For angles, the parasagittal error is the numerical difference, in degrees, of the morphometric measurement at a particular parasagittal slice and the morphometric measurement at the midsagittal slice. Estimating the parasagittal error would help show the importance of precise identification of the midsagittal plane to obtain morphometric measurements and understand the impact of inadvertent midsagittal plane selection. The objective of this study was to determine the absolute parasagittal error at planes positioned at various lateral distances from the midsagittal plane for morphometric parameters commonly examined in CMI research.

## Materials and methods

### Participants

A total of 30 adult female CMI subjects were involved in this study, ranging from 23 to 55 years of age (38.6 ± 7.3). MRI slices evaluated were taken before any surgical procedures were performed. The MRI images were obtained from the Chiari 1000 database collected by the Conquer Chiari Research Center in Akron, Ohio. More details on this database can be found in Houston et al. [9]. This study was approved by the University of Akron IRB (#201504145) and Akron General Medical Center (#14018). Electronic online consent was obtained from participants in the Chiari 1000 project. This consent procedure was approved by the University of Akron IRB.

### Imaging protocol

The MR images used in this study had an average slice thickness of 4.3 mm and were performed either on 1.5T or 3T scanners. Scans were performed on magnets from the following vendors: General Electric (Fairfield, CT), Philips (Amsterdam, Netherlands), Siemens (Munich, Germany), and Toshiba (Minato, Tokyo, Japan).

### Slicing and measurement protocol

**Fig 2:**
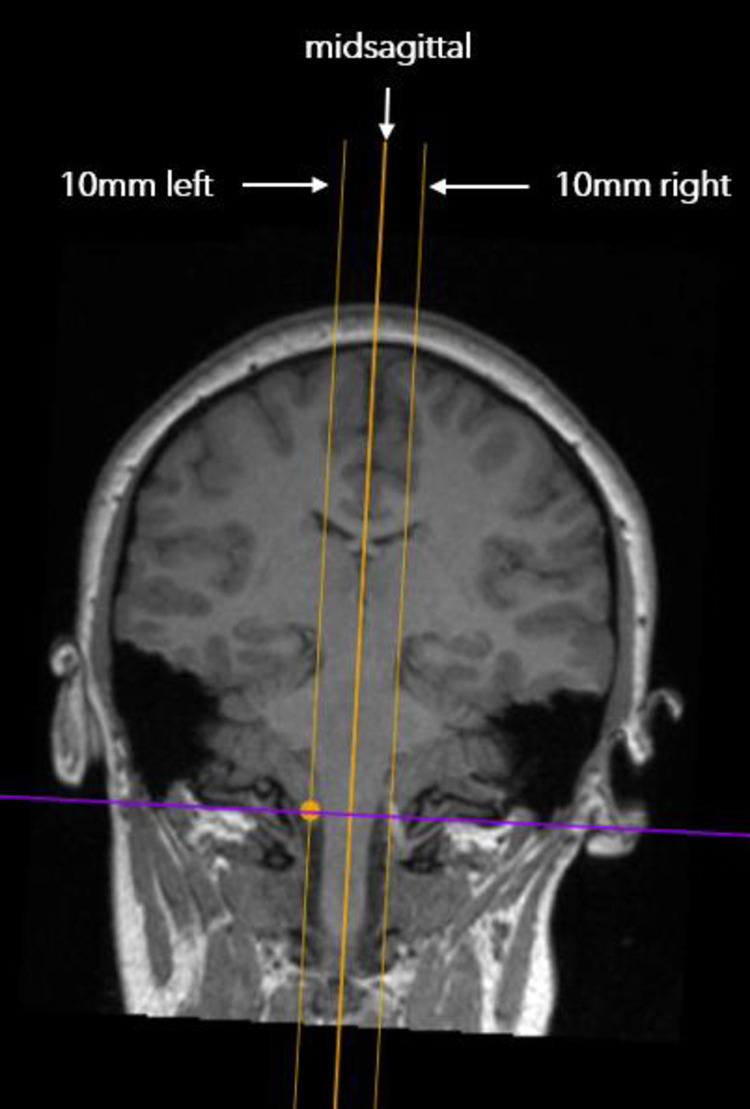
Coronal view of the locations of midsagittal, left and right parasagittal planes.

**Fig 3:**
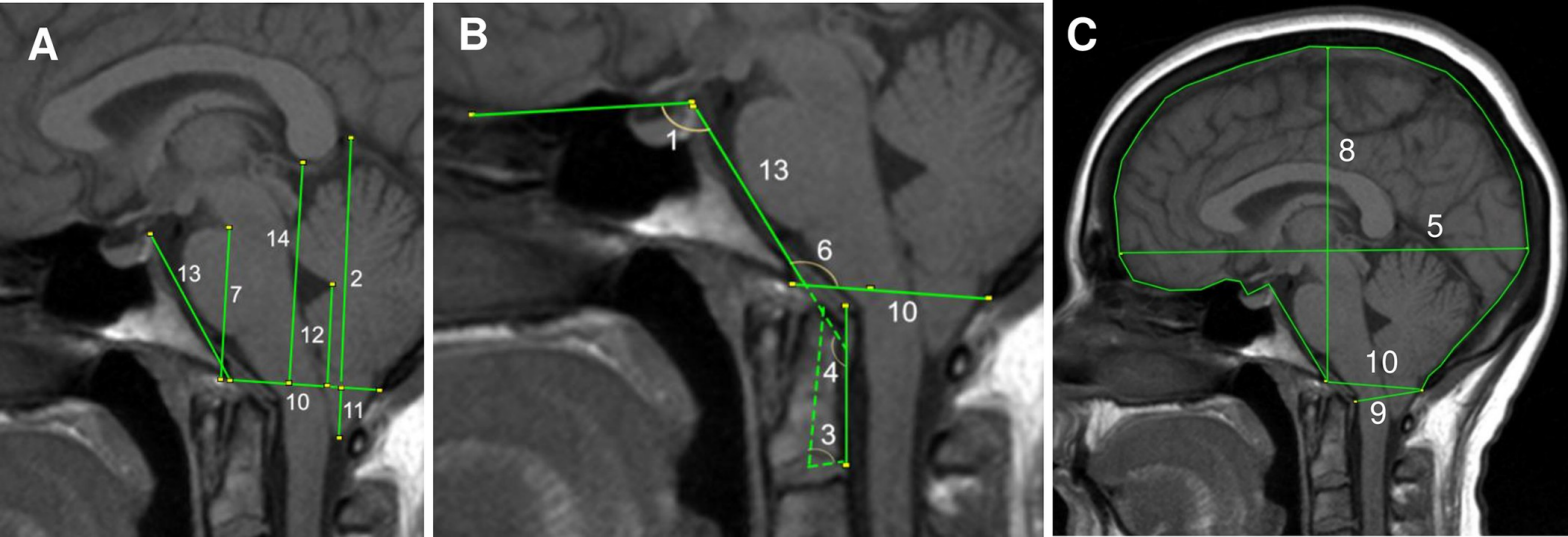
Fourteen morphometric measurements taken on a midsagittal T1-weighted MRI. **A:** 2-Posterior cranial fossa height, 7-pons height, 10- McRae line length, 11-tonsillar position, 12- fastigium height, 13- clivus length, 14-corpus callosum height. **B:** 1-Basal angle, 3-odontoid angle, 4-Wackenheim angle, 6- Boogard angle. **C:** 5-intracranial diameter, 8- intracranial height, 9-anteroposterior diameter dura-opisthion.

MRI scans were imported into Horos, a DICOM medical image viewer (Nimble LLC, Annapolis, MD USA). The scans were sliced using a slicing tool that allowed slices at specified intervals along the coronal plane to be obtained. Brain MRI imaging data of Five CMI subjects were sliced to obtain slices at 21 sagittal planes, the midsagittal plane, and 20 parasagittal planes located at 1mm lateral intervals (left and right) to the midsagittal plane (-10 mm to +10 mm, Fig 2). MRI imaging data of five CMI individuals were initially evaluated due to the time requirements to measure morphometrics on 21 sagittal slice per subject. The time required to measure all morphometric parameters on one slice, is approximately 25 minutes. To evaluate 21 slices per subject, the total time is approximately 525 minutes per subject. Fourteen morphometric parameters were measured on each of the 21 slices, using a custom in-house software developed in MATLAB (MathWorks, Natick, MA) at the University of Akron (see Appendix A for parameter descriptions, Fig 3) [8, 14]. The 14 morphometric parameters were selected because they were significantly different between CMI subjects and controls, as reported by Houston et al. [9]. To minimize the impact of human measurement variability, an extensive quality check was held for each slice evaluated, where another operator (DL) quality checked each slice and flagged any possible morphometric measurement discrepancies, and these were re-measured by the original operator (MM) until they passed the quality check.

### Statistical analysis

A series of 14 single-factor repeated-measures analyses of variance (ANOVAs), one for each of the 14 parameters, were computed using SAS software (v9.4) for group differences from the midsagittal plane to parasagittal planes 1 and 2 mm lateral. We also used Bonferroni correction to control for multiple comparisons. Therefore, the critical significance value was p< 0.0083 (0.05/6). For each morphometric parameter, we compared the midsagittal measurement to the other four measurements (left 1&2 mm, right 1&2 mm)–the five levels of the independent variable. The dependent variable was the morphometric parameter value at each of the five locations.

## Results

### Twenty-one sagittal planes (n = 5)

**Fig 4:**
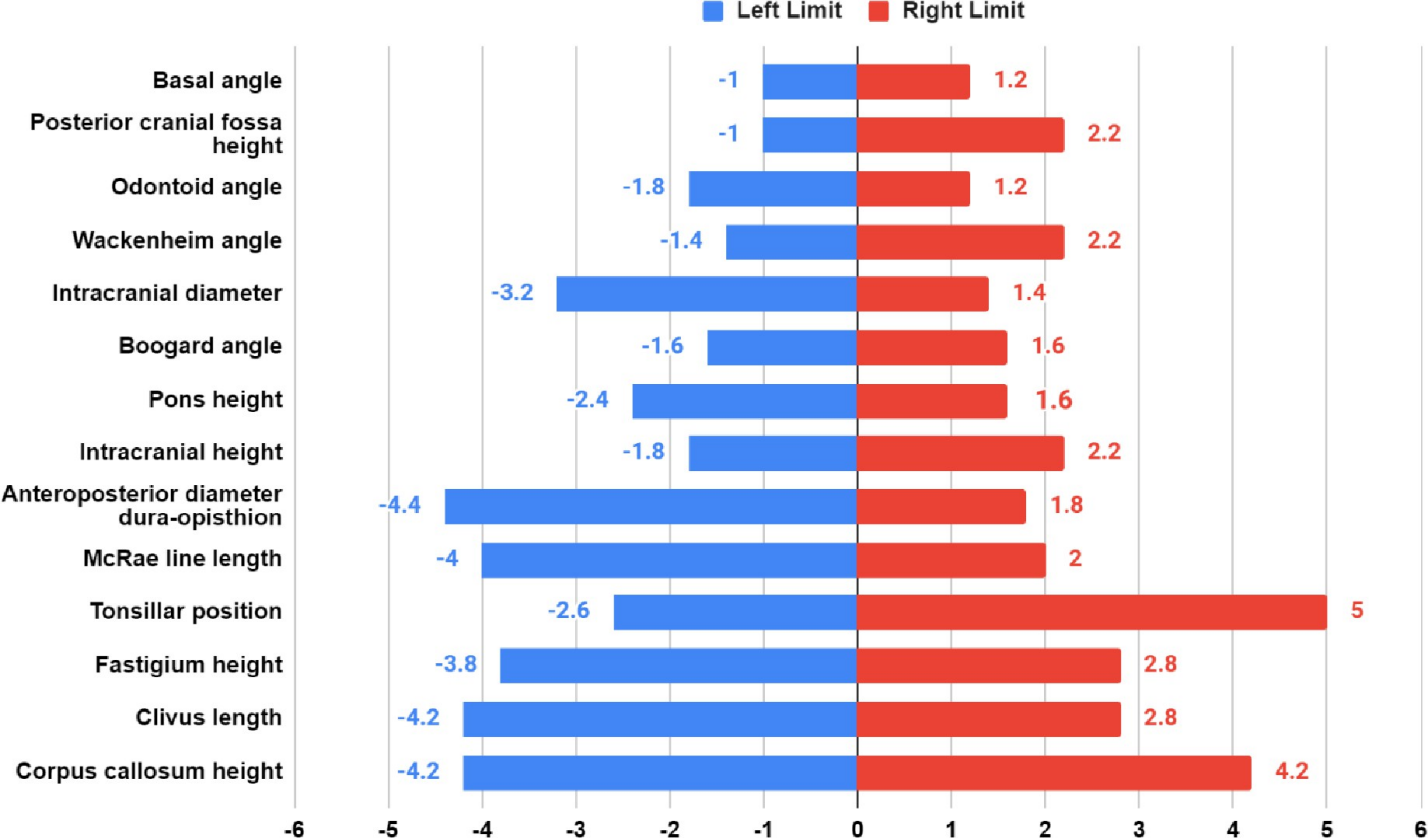
The average lateral distance from midsagittal plane (mm) at which the parasagittal error (difference between morphometric measurement at the parasagittal and midsagittal planes) exceeds 1 unit (mm/deg).

The results obtained from analyzing the MRI imaging data of five subjects demonstrated that for most of the parameters, the lateral distance at which the parasagittal error exceeds 1 unit (1 mm for lengths and 1 degree for angles) was approximately 2 mm (Fig 4). For each subject, the parasagittal distance at which the absolute parasagittal error exceeded 1 unit was identified for each morphometric parameter on both left and right sides of the midsagittal plane. An average value was found using the data for five subjects, for each morphometric parameter. The data on the left is the average lateral distance at which the parasagittal error exceeds 1 unit on the left side of the midsagittal plane, and the data on the right is the average lateral distance at which the parasagittal error exceeds 1 unit on the right side of the midsagittal plane.

**Fig 5:**
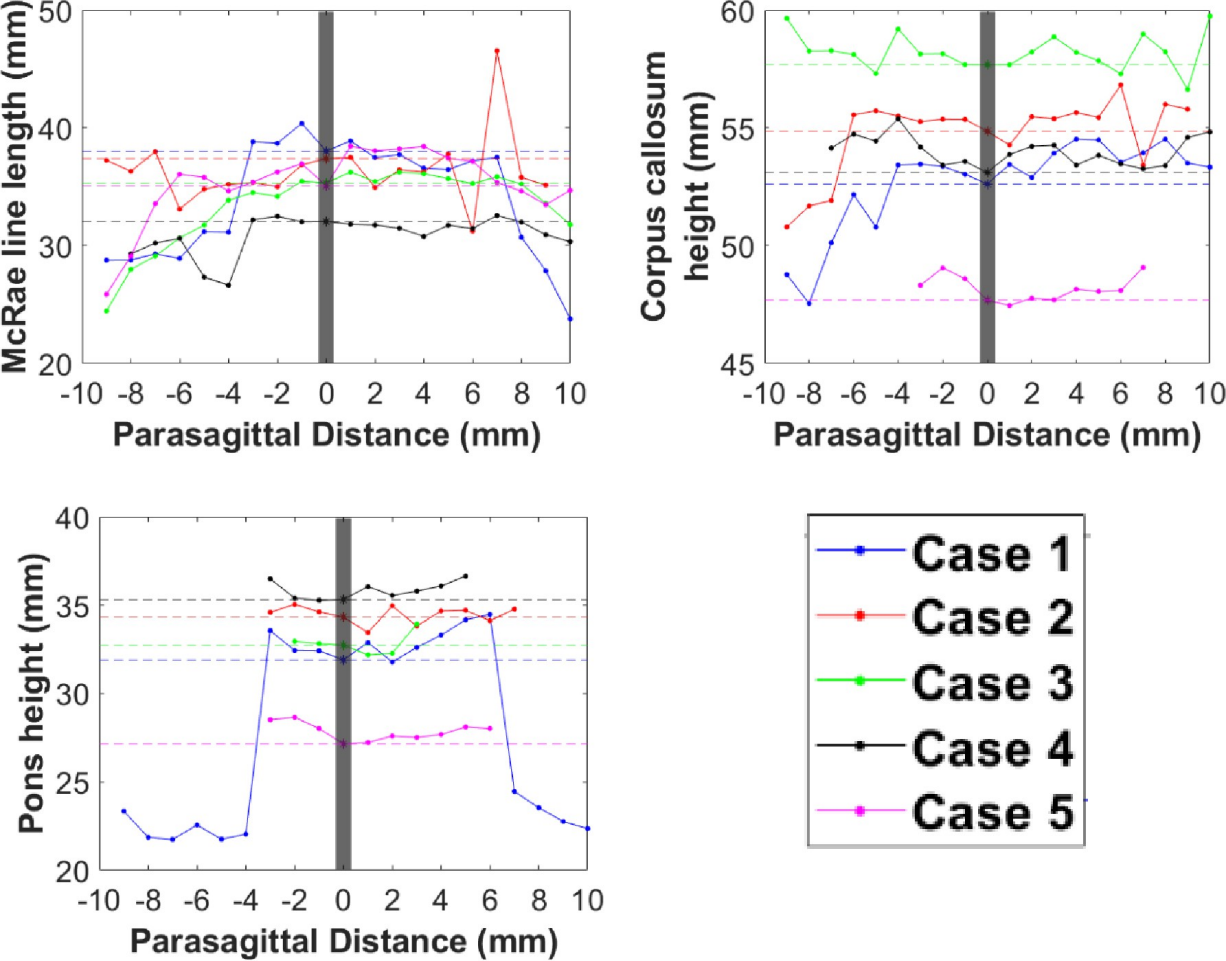
Symmetry plots showing parameters that maintain lateral symmetry up to 4 mm from the midsagittal plane.

**Fig 6:**
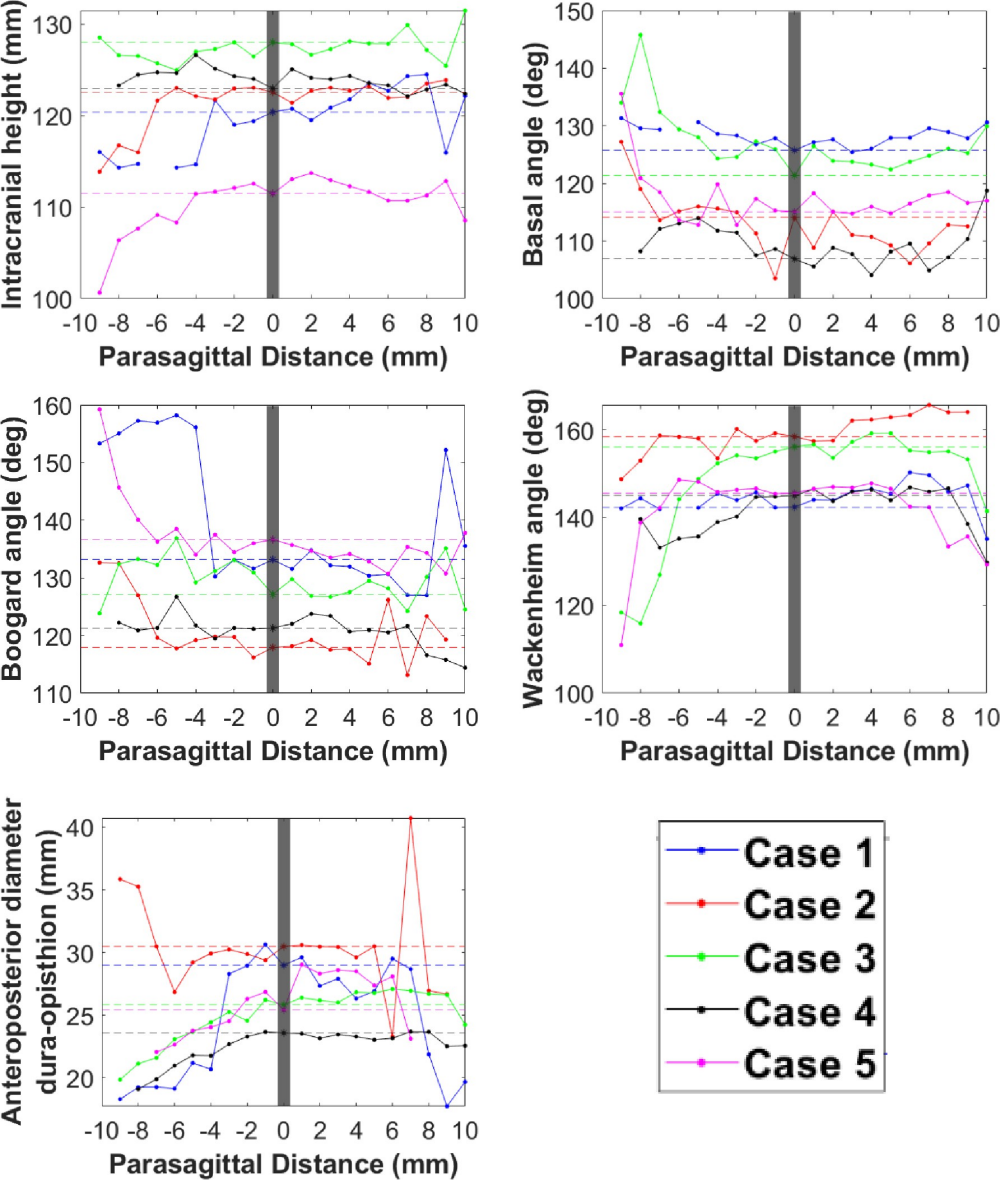
Symmetry plots showing parameters that maintain lateral symmetry up to 3 mm from the midsagittal plane.

**Fig 7:**
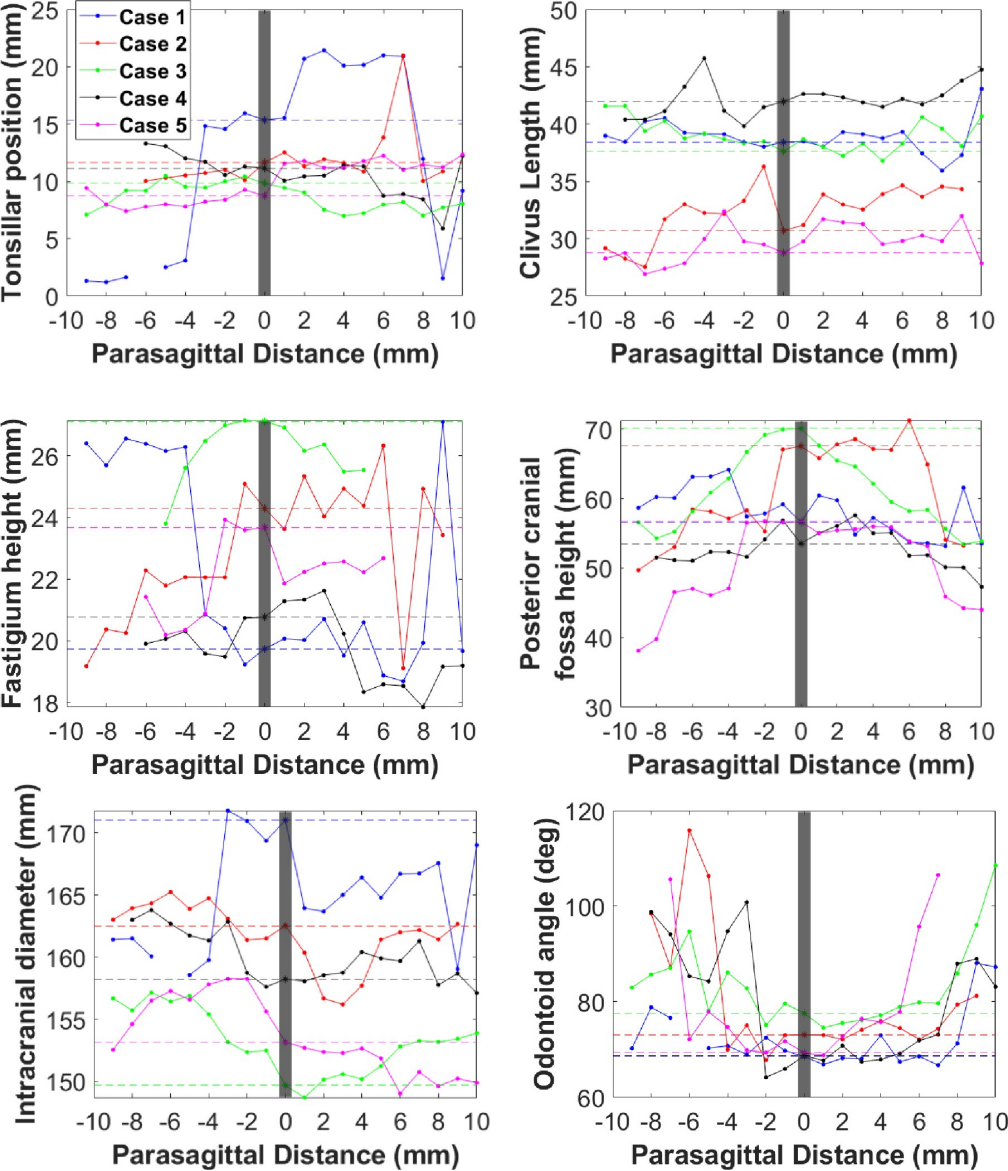
Symmetry plots showing parameters that demonstrate large lateral asymmetry from the midsagittal plane.

Symmetry plots generated using MATLAB (Figs 5–7) demonstrated that McRae line length, corpus callosum height, and pons height maintain lateral symmetry approximately up to 4 mm (Fig 5). Parameters such as anteroposterior diameter dura-opisthion, basal angle, intracranial height, Wackenheim angle, and Boogard angle maintain lateral symmetry approximately up to 3 mm (Fig 6). Tonsillar position, fastigium height, clivus length, posterior cranial fossa height, odontoid angle, and intracranial diameter demonstrated large lateral asymmetry (Fig 7).

The average lateral distance, at which the parasagittal error exceeds 1 unit (1 mm for lengths and 1 deg for angles), was calculated for each of the 14 morphometric parameters on 5/25 CMI subjects (Fig 4). As shown in Fig 4, on the right and left sides, 12/14 and 8/14 parameters exceed 1 unit difference at a lateral distance < 3 mm, respectively. Tonsillar position, corpus callosum height, clivus length, and fastigium height vary the least away from the midsagittal plane. Pons height, basal angle, Wackenheim angle, posterior cranial fossa height, odontoid angle, Boogard angle, and intracranial height vary the most away from the midsagittal. This result showed that the typical lateral distance at which the parasagittal error exceeded 1 unit for most of the parameters was approximately 2 mm.

### Five sagittal planes (n = 25)

Based on the results of the initial set of five subjects, an additional sample of 25 CMI subjects were evaluated at five sagittal planes (1 midsagittal and 4 parasagittal planes), the midsagittal and four parasagittal planes located 1 and 2 mm lateral (left and right) to the midsagittal plane. The mean individual absolute parasagittal error (MIAPE) is calculated as the mean value of the absolute parasagittal error (unsigned difference between midsagittal and parasagittal morphometric measurement) for a given parameter. The mean group parasagittal error (MGPE) is calculated as the mean value of a morphometric parameter at a particular lateral distance minus the mean value of the morphometric parameter at the midsagittal plane.

Table 1 shows the MIAPE for the 25 CMI subjects evaluated at 1 and 2mm lateral to the midsagittal plane to be > 1 unit for 9/14 parameters within a lateral distance of 2 mm. Table 2 shows the MGPE for the 25 CMI subjects evaluated at 1 and 2mm lateral to the midsagittal plane to be < 1 unit for 14/14 parameters within a lateral distance of 2 mm. Nine out of fourteen parameters had error < 0.5 unit, for example, the tonsillar position had a maximum parasagittal error of 0.33 mm. Using ANOVA, a comparison of the four parasagittal slice measurements (left 1&2 mm, right 1&2 mm) with the midsagittal measurements for all 14 parameters demonstrated that two of the 14 parameters were different (p<0.05 for corpus callosum height and anteroposterior diameter dura-opisthion). However, after Bonferroni correction (p<0.0083), none of the 14 parameters were significantly different (Table 2).

**Table 1:** Mean individual absolute differences between parasagittal and midsagittal plane measurements (MIAPE) on a given individual (n = 25).

Parameter	Left 2 mm	Left 1 mm	Right 1 mm	Right 2 mm
Basal angle (deg)	2.06 ± 2.81	2.02 ± 2.51	2.33 ± 3.20	2.19 ± 2.80
Boogard angle (deg)	1.55 ± 2.06	1.21 ± 1.64	1.24 ± 1.56	1.86 ± 2.41
Odontoid angle (deg)	2.99 ± 3.61	2.15 ± 2.67	2.91 ± 3.39	4.34 ± 7.72
Wackenheim angle (deg)	2.16 ± 3.01	1.72 ± 2.12	1.92 ± 2.85	1.89 ± 2.59
Intracranial diameter (mm)	1.88 ± 2.53	1.60 ± 2.10	1.42 ± 1.64	1.88 ± 2.37
Pons height (mm)	0.60 ± 0.76	0.41 ± 0.57	0.49 ± 0.61	0.69 ± 0.96
Intracranial height (mm)	0.93 ± 1.10	1.09 ± 1.64	0.60 ± 0.79	0.95 ± 1.19
Anteroposterior diameter dura-opisthion (mm)	1.13 ± 1.49	0.57 ± 0.67	0.49 ± 0.62	0.77 ± 0.96
McRae line length (mm)	0.74 ± 0.95	0.65 ± 0.83	0.57 ± 0.72	0.77 ± 0.94
Tonsillar position (mm)	1.28 ± 1.99	0.62 ± 1.01	0.52 ± 0.67	0.77 ± 1.03
Fastigium height (mm)	0.62 ± 0.74	0.45 ± 0.60	0.38 ± 0.49	0.57 ± 0.83
Clivus length (mm)	0.90 ± 1.20	0.53 ± 0.8	0.48 ± 0.65	0.76 ± 1.16
Corpus callosum height (mm)	0.40 ± 0.48	0.39 ± 0.5	0.37 ± 0.44	0.52 ± 0.67
Posterior cranial fossa height (mm)	1.18 ± 1.73	0.96 ± 1.16	1.02 ± 1.11	1.32 ± 1.61

**Table 2:** ANOVA results for group differences from the midsagittal plane to parasagittal planes 1 and 2 mm lateral (n = 25). *Note critical value is p< 0.0083 after Bonferroni correction. Differences from midsagittal measurements (MGPE) are given in parentheses. D = degrees of freedom.

Parameter	Mean Values					Maximum difference	D	F-value	p-value
	Left 2 mm	Left 1 mm	midsagittal	Right 1 mm	Right 2 mm				
Basal angle (deg)	116.53 (-0.81)	116.94 (-0.40)	117.34	116.75 (-0.59)	116.78 (-0.56)	0.81	4,96	0.65	0.58
Boogard angle (deg)	120.11 (+0.09)	120.15 (+0.13)	120.02	120.25 (+0.23)	120.08 (+0.06)	0.23	4,96	0.1	0.97
Odontoid angle (deg)	69.58 (-0.29)	69.95 (+0.08)	69.87	69.65 (-0.22)	70.59 (+0.72)	0.72	4,96	0.26	0.77
Wackenheim angle (deg)	151.66 (-0.21)	151.65 (-0.22)	151.87	151.91 (+0.04)	152.08 (+0.21)	0.22	4,96	0.21	0.89
Intracranial diameter (mm)	163.77 (+0.18)	163.36 (-0.23)	163.59	164.08 (+0.49)	164.4 (+0.81)	0.81	4,96	1.69	0.18
Pons height (mm)	38.29 (+0.31)	38.01 (+0.03)	37.98	38 (+0.02)	38.14 (+0.16)	0.31	4,96	1.36	0.26
Intracranial height (mm)	126.63 (+0.14)	126.78 (+0.29)	126.49	126.48 (-0.01)	126.68 (+0.19)	0.29	4,96	0.47	0.69
Anteroposterior diameter dura-opisthion (mm)	26.76 (-0.90)	27.51 (-0.15)	27.66	27.56 (-0.10)	27.47 (-0.19)	0.9	4,96	4.28	0.018
McRae line length (mm)	34.93 (-0.49)	35.36 (-0.06)	35.42	35.37 (-0.05)	35.53 (+0.11)	0.49	4,96	2.69	0.053
Tonsillar position (mm)	7.51 (+0.18)	7.23 (-0.10)	7.33	7.21 (-0.12)	7.00 (-0.33)	0.33	4,96	0.81	0.45
Fastigium height (mm)	27.13 (+0.21)	26.88 (-0.04)	26.92	26.94 (+0.02)	26.83 (-0.09)	0.21	4,96	1.39	0.25
Clivus length (mm)	41.22 (+0.23)	41.08 (+0.09)	40.99	40.91 (-0.08)	41.02 (+0.03)	0.23	4,96	0.59	0.61
Corpus callosum height (mm)	57.74 (+0.24)	57.50 (0)	57.5	57.50 (0)	57.33 (-0.17)	0.24	4,96	3.04	0.037
Posterior cranial fossa height (mm)	61.53 (-0.34)	61.75 (-0.12)	61.87	61.26 (-0.61)	61.08 (-0.79)	0.79	4,96	2.44	0.063

## Discussion

A comprehensive assessment of the parasagittal errors in the 14 morphometric parameters reveals that absolute errors may impact the clinical evaluation of an individual if clinicians consider that an error >1 unit (1 mm or 1 degree) is important. The current process of diagnosis of CMI utilizes patient-reported symptoms, and neurological signs and imaging confirmation of the cerebellar tonsils (≥ 5 mm below the foramen magnum). As shown in our results, the parasagittal error in an individual patient might impact the imaging component of the diagnosis of CMI. However, the impact of parasagittal errors for group measurements herein was minimal, which is relevant for research studies that evaluate the mean over a large number of subjects. Our results show the parasagittal error increased with increasing lateral distance from the midsagittal plane. Therefore, when thicker MRI sections are being evaluated, the greater risk of imprecise selection of the midsagittal plane could lead to errors in various morphometric measurements for an individual patient. For that reason, we recommend using thinner sectioned MRI scans in clinical assessments as this could potentially reduce the risk of errors from imprecise midsagittal plane selection.

In addition, this study revealed large asymmetry for morphometric parameters in the five CMI subjects evaluated at 21 sagittal planes. In particular, tonsillar position, fastigium height, clivus length, posterior cranial fossa height, odontoid angle, and intracranial diameter demonstrated large lateral asymmetry on an individual basis. In contrast, when averaged over all 25 CMI subjects, the asymmetry was not significant for morphometric parameters within a lateral distance of 2 mm.

Finally, the parasagittal errors obtained herein were compared to differences between adult female CMI subjects and healthy controls previously published [9]. For each parameter, the MIAPE and MGPE were compared to mean group differences between Chiari subjects and healthy controls (MGDCM) previously published, as shown in Table 3 [9]. The results demonstrate a similar trend as previously described. The MIAPE was relatively large compared to the MGDCM, indicating that a precise midsagittal plane is important on an individual basis. In contrast, the MGPE was relatively small compared to the MGDCM, even at a lateral distance of 2 mm. Thus, mean group measurements may not require a precise midsagittal plane.

**Table 3:** Comparison between maximum mean individual absolute parasagittal error (MIAPE), maximum mean group parasagittal error (MGPE), mean group differences between Chiari subjects and healthy controls (MGDCM) for each morphometric parameter.

Parameter	MGDCM	Maximum MIAPE		Maximum MGPE	
		within ± 2 mm	within ±1 mm	within ± 2 mm	within ±1 mm
Basal angle (deg)	4.1	2.19	2.33	0.81	0.59
Boogard angle (deg)	3.3	1.86	1.24	0.23	0.23
Odontoid angle (deg)	3.2	4.34	2.91	0.72	0.22
Wackenheim angle (deg)	6.0	2.16	1.92	0.22	0.22
Intracranial diameter (mm)	3.0	1.88	1.6	0.81	0.49
Pons height (mm)	2.3	0.69	0.49	0.31	0.03
Intracranial height (mm)	2.3	1.09	1.09	0.29	0.29
Anteroposterior diameter dura-opisthion (mm)	1.8	1.13	0.57	0.9	0.15
McRae line length (mm)	1.3	0.77	0.65	0.49	0.06
Tonsillar position (mm)	10.9	1.28	0.62	0.33	0.12
Fastigium height (mm)	3.0	0.62	0.45	0.21	0.04
Clivus length (mm)	3.0	0.9	0.53	0.23	0.09
Corpus callosum height (mm)	2.9	0.52	0.39	0.24	0.00
Posterior cranial fossa height (mm)	2.6	1.32	1.02	0.79	0.61

## Conclusion

Midsagittal morphometric measurements on an individual basis can have substantial parasagittal errors with imprecise midsagittal plane selection, whereas mean group error is relatively small within ±2 mm off the midsagittal plane. Thus, imprecise midsagittal plane selection may impact the clinical assessment of an individual patient. However, its impact on group measurements in a research setting will be minimal.

## Limitations

Some limitations should be considered in this study. The sample size, consisting of a total of 30 subjects, is small. This sample size was selected to be able to obtain sufficient data for statistical analysis, despite the significant time required to evaluate the many individual slices for each subject. In addition, the MRI scans obtained through the Chiari 1000 project had a relatively large slice thickness (average 4.3 mm), which reduces the quality of the MRI sequences sliced, potentially limiting the accuracy of morphometric measurements obtained.

## Supporting information

S1 Appendix Parameter descriptions for 14 parameters assessed. (PDF)

S1 Table Raw data showing 14 morphometric parameter measurements for each subject (n = 5) evaluated at twenty-one sagittal planes (1 midsagittal and 20 parasagittal planes). (PDF)

S2 Table Raw data showing 14 morphometric parameter measurements for each subject (n = 25) evaluated at five sagittal planes (1 midsagittal and 4 parasagittal planes). (PDF)
