# Supplementary material for: The importance of precise plane selection for female adult Chiari Type I malformation midsagittal morphometrics
Source: PLoS One. 2022 Aug 10;17(8):e0272725. doi: 10.1371/journal.pone.0272725 (PMC9365159; doi:10.1371/journal.pone.0272725)
Supplement: S1 Appendix — (PDF) [file pone.0272725.s001.pdf]

Parameter	Description
1- Basal angle	A line extending across the anterior cranial fossa to the tip of the dorsum sellae, and the line drawn along the posterior margin of the clivus
2- Boogard angle	Constituted by the clivus length and the McRae line
3- Odontoid angle	The angle formed by the top of the odontoid process, with the vertex as the midpoint between the anterior-inferior and the posterior-inferior corticated portion of the odontoid, and the posterior-inferior base of the odontoid process.
4- Wackenheim angle	Formed by a line along the clivus and a line tangent to the posterior aspect of the odontoid process until the base of the C2 vertebra
5- Intracranial diameter	The maximum length of the skull parallel to the plane of foramen magnum
6- Pons height	A perpendicular line from the cephalad aspect of the pons at the mid-brain junction to the McRae line
7- Intracranial height	The maximum length of the skull perpendicular to the plane of foramen magnum
8- Anteroposterior diameter dura-opisthion	The distance between the vertex of Dural angle and the opisthion
9- McRae line length	The distance from the basion to the opisthion
10- Tonsillar position	The perpendicular distance between the tip of the cerebellar tonsil and the McRae line
11- Fastigium height	A perpendicular line from the fastigium of the fourth ventricle to the McRae line
12- Clivus length	The distance between the dorsum sellae and the most inferior point of the clivus (basion)
13- Corpus callosum height	A perpendicular line from the inferior-most aspect of the splenium of the corpus callosum to the McRae line
14- Posterior cranial fossa height	The perpendicular distance from the most anterior portion of the tentorium to the McRae line
