# Supplementary material for: The importance of precise plane selection for female adult Chiari Type I malformation midsagittal morphometrics
Source: PLoS One. 2022 Aug 10;17(8):e0272725. doi: 10.1371/journal.pone.0272725 (PMC9365159; doi:10.1371/journal.pone.0272725)
Supplement: S1 Table — (PDF) [file pone.0272725.s002.pdf]

	McRae line length	Tonsillar Position	Fastigium height	Pons height	Corpus Callosum height	Clivus length	Basal angle	Wackenheim angle	Posterior cranial fossa height	anteroposterior diameter dura-opisthion	Odontoid angle	Intracranial height	Intracranial diameter	Boogard angle
**2559-mid**	38.04386473	15.34377211	19.73880488	31.91391636	52.62530012	38.41822036	125.7485345	142.4491998	56.63920185	28.98589858	68.53095191	120.401931	171.0268165	133.1270057
2559-1L	38.89980469	15.5375811	20.07034025	32.88830108	53.45306744	38.49770465	127.1539322	144.0951616	60.44527558	29.62519336	66.88068849	120.7456876	163.9431465	131.5590607
2559-1R	40.39135934	15.94574459	19.22877885	32.4229156	53.0334031	38.02510496	127.851562	142.3282618	59.18023522	30.62888748	69.77763998	119.3967519	169.3704124	131.6353865
2559-2L	37.49353052	20.68527686	20.02708414	31.79225363	52.89840722	38.03825694	127.6290674	144.0034213	59.75851222	27.34177794	68.20799163	119.4974671	163.6749032	134.78783
2559-2R	38.70616244	14.56520754	20.40679848	32.43753278	53.37615722	38.43609639	126.7786981	145.7672594	57.85847621	28.95478	72.52656504	119.0094892	170.9605877	133.1322332
2559-3L	37.74407442	21.42585991	20.70526282	32.61123713	53.93102573	39.30528767	125.4214334	146.0543125	54.85833318	27.90312264	68.09668326	120.8595212	165.0050019	132.1671673
2559-3R	38.83455401	14.82363963	20.85888571	33.56238502	53.4630887	39.109976	128.3141761	144.0231297	57.43672594	28.29368152	68.96606393	121.6804464	171.7810522	130.2426991
2559-4L	36.58554035	20.104877	19.52229685	33.31082667	54.52878705	39.11219183	126.0431947	146.5687622	57.23228103	26.3238725	73.04264577	121.7634233	166.4105747	131.9528856
2559-4R	31.16203631	3.105877789	26.28688478	22.04630952	53.42586807	39.14665217	128.6112085	145.5400576	64.15923186	20.68101313	70.78992749	114.6667131	159.7711688	156.1255575
2559-5L	36.47388913	20.15613175	20.60054379	34.17267746	54.49607963	38.76512217	127.9190234	145.4992066	55.62711308	26.91556599	67.44912452	123.5635372	164.789492	130.3693488
2559-5R	31.20248325	2.51477993	26.15901236	21.76127123	50.79487624	39.24684798	130.6327731	142.2676371	63.19901469	21.17986185	70.32273986	114.3100954	158.5684577	158.2166537
2559-6L	37.20912071	20.98218915	18.87657609	34.48652462	53.55320427	39.32678271	127.9493073	150.3304922	53.75403616	29.50558522	68.5935006	122.6898138	166.7097057	130.6408355
2559-6R	28.93131608		26.39420453	22.56596204	52.17008428	40.51827977			63.14759797	19.13353596				156.9292588
2559-7L	37.51464951	20.89958222	18.68692193	24.4689	53.94868095	37.44641964	129.5667905	149.7049481	53.60001933	28.67970793	66.76296111	124.3112783	166.7375475	127.0381486
2559-7R	29.30815689	1.639253699	26.5638952	21.74661754	50.12605185	40.24946113	129.3697505	141.9744436	60.09703377	19.25521542	76.6535944	114.7294354	160.0623576	157.2526903
2559-8L	30.71729566	11.95862817	19.93383853	23.54470868	54.53086341	35.93670966	128.9305763	145.8690562	53.16991706	21.87545116	71.29206053	124.4854838	167.5644421	126.9868907
2559-8R	28.80248791	1.216880508	25.69765308	21.86805854	47.54781632	38.45026368	129.5667905	144.4360491	60.25348045	19.23964465	78.85464281	114.3151865	161.5094099	155.0749318
2559-9L	27.87673083	1.537985576	27.10476896	22.768	53.50883406	37.30061813	127.8580529	147.3359446	61.58474288	17.72536855	88.10569392	115.9457041	159.0456516	152.1804972
2559-9R	28.78971851	1.321844633	26.4080943	23.34970868	48.77865881	38.97844992	131.3308707	142.1142593	58.68241413	18.28818134	70.25916647	116.0113054	161.4109454	153.3024295
2559-10L	23.79799608	9.174157356	19.66944959	22.3589	53.34082069	43.06367017	130.5926601	135.1840277	53.57272202	19.67190987	87.31622484	122.1417304	169.0183239	135.5222988
2559-10R	30.14177919	4.642214504	25.06741474	27.58421253	50.93661711	38.50511305	136.3873247	137.7461085	56.63882204	19.88162004	93.48528695	119.1456424	171.2908383	145.7760039
**2597-mid**	37.39030862	11.63173821	24.29390176	34.32894486	54.86009226	30.708757	114.093663	158.5217373	67.55629388	30.48502343	73.12567138	122.5504755	162.5393556	117.9232836
2597-1L	37.5090499	12.52714791	23.63055062	33.45801219	54.27726932	31.19398155	108.864344	157.483707	65.83313343	30.59890724	73.06779368	121.3984095	160.3702999	118.1757994
2597-1R	36.86628918	10.11020727	25.09442026	34.63056228	55.36551686	36.29895653	103.5387111	159.2746668	67.09299409	29.39118339	73.05860975	123.0386296	161.5085414	116.1861663
2597-2L	34.95436622	11.32675226	25.33994982	34.96965493	55.47837143	33.86386965	115.1280369	157.5975753	67.81854696	30.46995445	72.153985	122.7028427	156.6904052	119.23335
2597-2R	35.02528941	11.01355878	22.06046596	35.04504037	55.37354421	33.31399548	111.3383658	157.5358849	55.29247983	29.88245151	67.8091034	122.9593277	161.3811012	119.7600711
2597-3L	36.45484561	11.92008597	24.03633791	33.8206167	55.39086798	32.98767224	111.0805853	162.1443195	68.56801759	30.44056115	74.16252298	123.0461132	156.1955096	117.5213175
2597-3R	35.36858054	10.73619909	22.05965171	34.60708695	55.27066709	32.16694101	114.9870304	160.2155381	58.31449031	30.24703793	75.09649097	121.7486268	163.1023346	119.8019109
2597-4L	36.2702544	11.60639937	24.93765859	34.68178359	55.65595627	32.55429542	110.7555302	162.3700857	67.16596038	29.62060853	75.9969959	122.7121901	157.713026	117.6539693
2597-4R	35.22023298	10.52340305	22.06516491		55.50489846	32.24998819	115.6461135	153.5525991	57.13014703	29.92849703	69.96992304	122.1172906	164.7391501	119.2089752
2597-5L	37.78677359	10.85030888	24.38280615	34.71799936	55.44617438	33.88429516	109.2747012	162.9169131	67.02880439	30.49724832	74.53317776	123.1704347	161.4213047	115.1032031
2597-5R	34.81031869	10.31905746	21.79340426		55.73207406	33.00895787	116.0131863	158.0750573	58.15283614	29.21078811	106.3649182	123.0192233	163.8842379	117.7490157
2597-6L	31.24658705	13.82373894	26.32928562	34.12241845	56.82790965	34.64430203	106.1561389	163.3870944	71.25890569	23.27786828	72.17262722	121.9352515	162.0082208	126.1807517
2597-6R	33.12069441	10.03612626	22.28545197		55.55733052	31.70773182	115.1732974	158.4690673	58.4582473	26.83703309	115.8991807	121.6316211	165.245791	119.6236087
2597-7L	46.55325132	20.97025395	19.11041039	34.78528793	53.42183172	33.65958569	109.608239	165.7175016	64.91972429	40.73905044	74.38901147	122.0330762	162.1891833	113.1341529
2597-7R	37.9955307		20.25508276		51.92508951	27.53369342	113.6396294	158.757556	53.03799531	30.48820669	87.27002226	115.9856824	164.3327449	126.9978969
2597-8L	35.80801208	10.03364534	24.93428683		56.0046267	34.55433406	112.8214439	164.0373758	54.08789868	26.95461715	79.42305893	123.4896446	161.4327346	123.3491778
2597-8R	36.33054831		20.37177168		51.68629425	28.25383719	119.0488452	153.0202414	51.51912758	35.25839895	98.47043135	116.74182	163.9405029	132.5359985
2597-9L	35.14604165	10.86529668	23.43576288		55.79488417	34.32458567	112.5699286	164.0888514	53.23604381	26.6815823	81.25833031	123.8756776	162.6755803	119.3243735
2597-9R	37.25254443		19.17644523		50.7994774	29.17159274	127.2267173	148.8095355	49.7092728	35.85310796		113.8751656	163.0117846	132.6401487
2597-10L	36.33095155	12.66939099	22.49069016		52.05012557	27.67893202	113.1730443	160.8207	51.26246841	24.39032744	76.94413843	116.4129672	159.3558426	129.595686
**11080-mid**	35.33351002	9.823415459	27.12634027	32.73772146	57.68108052	37.61495569	121.4008238	156.1966132	70.10028454	25.81671837	77.61113307	127.9912671	149.6862221	127.1683105
11080-1L	36.25008834	9.444091194	26.92057017	32.19092956	57.68342875	38.68419866	126.4722115	156.6953748	67.63900669	26.3934508	74.5894955	127.7980313	148.7146112	129.7644443
11080-1R	35.4864077	10.42201616	27.1515814	32.83074422	57.68892474	38.48621935	125.9366604	155.0867956	69.95708681	26.2129935	79.63774135	126.4533316	152.4890049	130.9322528
11080-2L	35.45574146	9.032323989	26.16403623	32.28041311	58.22690379	38.00269844	123.9228872	153.6571835	65.47284411	26.17429714	75.60566257	126.6407964	150.1449022	126.8598057
11080-2R	34.19209805	10.01396824	26.99505546	32.95282429	58.15187274	38.31432597	127.3028606	153.5676941	69.18820568	24.55338524	75.1624692	127.9965898	152.3553798	133.1035829
11080-3L	36.25008834	7.533176728	26.36816449	33.94226016	58.8659861	37.22981512	123.7611165	157.3036553	64.62328086	26.00848244	76.18788047	127.2497175	150.5895675	126.7200211
11080-3R	34.51950921	9.467798998	26.48405499		58.13887463	38.68355958	124.6039398	154.2122667	66.71861987	25.26010174	82.82502828	127.2638337	153.168933	131.2018911
11080-4L	36.10864204	6.99579265	25.48966142		58.20926709	38.28850769	123.2644969	159.2695586	62.15698097	26.83191639	77.19301111	128.1096827	150.1907784	127.546104
11080-4R	33.86882137	9.541664503	25.6118363		59.19452452	39.18644284	124.3390374	152.3876015	62.90841596	24.4363099	86.16513927	126.9788829	155.4088827	129.2185201
11080-5L	35.70241119	7.226345291	25.54287324		57.85230897	36.79700431	122.4614549	159.2804109	59.5252536	26.7756543	78.96802782	127.8722434	151.2451991	129.4808806
11080-5R	31.76943629	10.49591331	23.8041213		57.3104258	38.74932899	128.0128854	148.8304853	60.87598591	23.65179893	78.16640687	124.9673569	156.8965273	136.8748914
11080-6L	35.28519953	7.981593126			57.29483492	38.28850769	123.7473537	155.3357559	58.22106966	27.08501868	79.9020643	127.8470176	152.8111104	128.1931472
11080-6R	30.65742866	9.179971801			58.1183176	40.27105553	129.4073028	144.2141819	58.17476508	23.08475027	94.74908507	125.7085211	156.4421015	132.2471406
11080-7L	35.86131966	8.185646091			58.98711415	40.59633032	124.809957	154.9358049	58.37908486	26.94959273	79.68174973	129.8949115	153.2705587	124.2373287
11080-7R	29.13506178	9.211621351			58.28370001	39.39282779	132.3997729	126.988989	55.24936343	21.59638237	87.10423803	126.5053615	157.1568599	133.2473784
11080-8L	35.23822595	7.025475725			58.23273288	39.59314221	126.0464802	155.1266141	55.67903489	26.69891055	85.96743366	127.1615316	153.1910674	130.1672023
11080-8R	28.00320131	7.94189152			58.26643401	41.57829218	145.7526456	115.9257964	54.30431649	21.12534235	85.72067012	126.5794138	155.7200052	132.3908755
11080-9L	33.6028116	7.723481985			56.6349441	38.06185069	125.2654103	153.3111483	53.44196336	26.61823079	96.08435327	125.4297006	153.4282546	135.1159008
11080-9R	24.46563126	7.099600414			59.65442784	41.5687777	133.9988196	118.4072527	56.59066632	19.85113084	83.0111198	128.5163079	156.6860768	123.852308
11080-10L	31.80054772	8.041482073			59.74827399	40.67967357	129.9294027	141.4995973	53.88352721	24.23822793	108.575134	131.4685921	153.8872917	124.5124705
11080-10R	28.56694873				48.1873626	31.57586119	135.8631857	117.5890941	47.26138153	18.18597916	92.18395023	111.3133095	152.6953568	155.7600827
**2861-mid**	32.06643311	11.14713275	20.7657465	35.33405924	53.11033146	41.96920814	106.9031641	145.042811	53.55256658	23.58045162	68.71300382	122.9729503	158.2477175	121.3195728
2861-1L	31.82878285	10.06551253	21.28319819	36.05341768	53.88009647	42.62765024	105.5940675	146.5409259	55.05627321	23.52735007	67.71899348	125.0587924	158.0720431	122.0224827
2861-1R	32.03612457	11.2970184	20.73910617	35.28663627	53.58347556	41.47429736	108.6243004	144.8486406	56.82960415	23.6545264	65.97740217	124.0059692	157.6207551	121.1382166
2861-2L	31.76775332	10.44064018	21.33752168	35.55646448	54.22159168	42.62091355	108.8419038	143.7986966	56.06824142	23.15213139	70.81354892	124.124527	158.5497684	123.7550705
2861-2R	32.51283011	10.55373165	19.48169262	35.40615079	53.42482203	39.82290995	107.5393238	144.7358149	54.15073553	23.28739153	64.23190208	124.3060122	158.7357816	121.3177418
2861-3L	31.48689943	10.51208398	21.62626854	35.80030853	54.26801663	42.32362345	107.7434624	145.9289632	57.6042461	23.45844633	67.45109354	123.9734974	158.7495836	123.3935849
2861-3R	32.20621318	11.70881209	19.57779691	36.49450017	54.18946426	41.16076509	111.4564656	140.2579445	51.62870692	22.68700489	100.8510388	125.1237513	162.8618271	119.4878871
2861-4L	30.79473386	11.40801607	20.23040912	36.08598772	53.42529937	41.88195735	104.1162605	146.4245015	55.02540363	23.28739153	67.93470483	124.3281012	160.420802	120.6779327
2861-4R	26.65358954	12.00898024	20.30804902		55.38300053	45.71888128	111.8264507	139.0059121	52.3130198	21.75955175	94.80739638	126.6008604	161.3405438	121.7563774
2861-5L	31.74904469	11.31810919	18.33987046	36.64635416	53.84228903	41.49624144	108.1885169	143.9881108	55.08814207	23.02775933	69.20158704	123.5327424	159.9077144	120.952938
2861-5R	27.34748336	13.06102619	20.0634889		54.44824253	43.26039679	114.014717	135.7099943	52.34340871	21.79380028	84.32636592	124.6550974	161.7456951	126.7326807
2861-6L	31.46524023	8.743842846	18.5887622		53.47327678	42.19479565	109.5556574	146.9278836	51.79017699	23.15264545	71.91078973	123.3025773	159.6939918	120.5506415
2861-6R	30.63542859	13.29676483	19.90635223		54.7388598	41.13272957	113.071517	135.2375249	51.0578447	20.9720908	85.39020161	124.7286704	162.6952963	121.3331736
2861-7L	32.58240537	8.883955228	18.53674684		53.26986192	41.72015129	104.9172255	145.9240062	51.86427999	23.68927453	73.15585151	122.1061204	161.3027158	121.6577817
2861-7R	30.22956777				54.1503481	40.41118502	112.1371543	133.1862775	51.1581591	19.89146368	94.1677743	124.4635364	163.7985978	120.9124497
2861-8L	32.01095248	8.433259564	17.84803715		53.40591939	42.49557645	107.181486	146.6778513	50.14068885	23.66410683	88.00048988	122.8456621	157.7788827	116.5842801
2861-8R	29.3174554					40.3924756	108.2458609	139.692246	51.53222831	19.09089812	98.8032077	123.2919896	162.9965123	122.2303343
2861-9L	30.93893054	5.894812512	19.16079121		54.59946397	43.7842324	110.3678418	138.5810569	50.09353714	22.51262852	89.00099335	123.3846458	158.6886243	115.7760056
2861-9R														
2861-10L	30.35140244	12.23982251	19.19177894		54.83104746	44.74335632	118.7443256	129.8007988	47.3170956	22.55320014	83.18807223	122.3854524	157.1220885	114.4130148
2861-10R	19.45127133					48.12308074	141.7034975	130.1970055	49.74942595	18.46477498	88.26951808	125.2774718	156.779483	118.8195779
**2880-mid**	35.08984915	8.728277083	23.67987011	27.15350686	47.68942981	28.7682716	115.0937207	145.6437059	56.67262153	25.43063962	69.3211772	111.4656725	153.1769307	136.6611838
2880-1L	38.46125372	11.55220252	21.86479603	27.23564049	47.46510616	29.77081898	118.3065957	146.5789541	55.02195346	29.03964103	68.83874018	113.0571104	152.7088914	135.713727
2880-1R	36.96933388	9.269787127	23.5934345	28.02660336	48.60491009	29.50170781	115.3044469	145.4620245	56.59750533	26.84965589	71.77339221	112.571932	155.6311248	136.0098016
2880-2L	38.07204742	11.78049008	22.23469982	27.60223438	47.77729885	31.70990218	115.0893221	147.0260983	55.45412153	28.31324965	72.82145341	113.73244	152.3655632	134.6810289
2880-2R	36.26515695	8.389378378	23.92969123	28.65935166	49.062964	29.77326352	117.3313988	146.6665535	56.73766396	26.2953549	69.38572306	112.103245	158.2559116	134.4442741
2880-3L	38.23396862	11.19674972	22.50779193	27.52178773	47.70771994	31.42447344	114.7835256	146.8671067	55.60799248	28.60228675	76.45705615	112.9609025	152.2914808	133.5212652
2880-3R	35.40874283	8.22948023	20.87894836	28.52235285	48.32682901	32.39011252	112.8051989	146.3661056	56.55630924	24.52338728	69.88782717	111.6962688	158.2639344	137.5054743
2880-4L	38.44284083	11.17823767	22.57099142	27.69051507	48.16080907	31.2905289	115.984553	147.8385223	55.98167515	28.49940094	75.77378149	112.2940951	152.6579352	134.1374264
2880-4R	34.65897157	7.804999991	20.35516399			29.9707494	119.8680322	145.8713733	47.07710147	24.04225785	74.75536159	111.4513099	157.8131779	134.0139869
2880-5L	37.44115989	11.77421816	22.22578008	28.11893052	48.06590762	29.53576655	114.8169501	146.5906981	55.89551701	27.37795295	77.86582092	111.6661791	151.860989	132.8106087
2880-5R	35.81312307	8.007978362	20.19518841			27.86039938	112.8206405	148.2024231	46.10126833	23.76511367	77.89591946	108.3264589	156.5882801	138.505441
2880-6L	37.16801428	12.23987924	22.67984689	28.02993999	48.10199682	29.80965949	116.5055521	142.5280419	53.86763889	28.09569695	95.77354014	110.7153805	149.0385958	130.7281547
2880-6R	36.06789714	7.795125863	21.42908476			27.38987088	113.63763	148.6305138	47.0311491	22.65083945	72.1932692	109.1517924	157.2749846	136.2938743
2880-7L	35.37298945	11.02360347			49.08303149	30.28783779	117.9234013	142.374152	53.16885035	23.12755207	106.5641655	110.7072804	150.7500215	135.3456819
2880-7R	33.57682316	7.412250313				26.92464836	118.4728921	142.2588885	46.5502212	22.06135786	105.6777922	107.6697087	156.5072602	140.0885732
2880-8L	34.65108545	11.44609101				29.79761706	118.5003175	133.4520391	45.90522338			111.2759108	149.627868	134.3233477
2880-8R	29.13611337	7.997384815				28.75216991	120.948275	138.9015571	39.80749557			106.3949519	154.6167463	145.6967789
2880-9L	33.49393834	11.23347547				31.98110865	116.6351297	135.7158239	44.24969143			112.8579744	150.2280017	130.7631549
2880-9R	25.88892341	9.422810899				28.26705305	135.5845136	110.9689149	38.13066117			100.6656339	152.5530679	159.2324689
2880-10L	34.70505666	12.34153731				27.85307088	116.9996095	129.3083924	44.01911113			108.5646259	149.8939835	137.8220927
2880-10R	26.63208979	8.289592636				29.47036982	136.3756393	117.6029567	38.30643052			101.2181556	152.8243256	159.8451985
