# Supplementary material for: The importance of precise plane selection for female adult Chiari Type I malformation midsagittal morphometrics
Source: PLoS One. 2022 Aug 10;17(8):e0272725. doi: 10.1371/journal.pone.0272725 (PMC9365159; doi:10.1371/journal.pone.0272725)
Supplement: S2 Table — (PDF) [file pone.0272725.s003.pdf]

	McRae line length	Tonsillar position	Fastigium height	Pons height	Corpus Callosum height	Clivus length	Basal angle	Wackenheim angle	Posterior cranial fossa height	Anteroposterior diameter dura-opisthion	Odontoid angle	Intracranial height	Intracranial diameter	Boogard angle
**2455-mid**	32.96947683	1.365599632	28.99948361	36.62245584	58.81344986	39.94410486	113.6955901	148.0588854	64.93540745	24.11378702	79.32784488	125.1811285	173.1322621	120.7376041
2455-1L	34.22031397	2.453629725	28.86825144	37.01886232	58.41034691	40.01333875	113.2118501	145.179757	65.09078373	25.4577629	81.35093046	124.2444241	172.1988458	119.3003737
2455-1R	33.00699276	0.751894991	29.38609057	36.40196346	59.51501753	40.33676344	111.5731996	142.4095541	63.51821215	23.16353649	81.35652763	124.7831131	173.881591	122.0294635
2455-2L	33.60441356	3.265524468	28.33029433	36.8728143	58.39689659	41.08714903	110.1655737	145.9665087	64.33831031	24.99447828	81.81737766	123.3874362	173.3849219	119.3513036
2455-2R	33.22690735	0.586820816	29.82405256	37.10129835	59.52688935	40.12607547	110.4455955	143.0676361	62.62617044	22.66455773	110.6859034	125.3820545	172.3071917	120.996122
**2488-mid**	39.1386058	5.560102688	28.97256149	40.71627899	59.72165305	40.23944547	124.7373207	155.3752233	63.62671485	29.88367829	73.52904782	132.6472828	167.66203	112.5496881
2488-1L	38.64932563	5.765905139	28.42413023	40.99867401	59.23847487	40.09059637	120.5594583	158.454622	64.33271562	29.15503319	72.6192374	131.413858	169.632735	113.9255205
2488-1R	39.07213535	5.681324001	28.4526149	40.99061961	59.32450198	41.00806138	115.5545614	161.8629468	64.15745254	29.72195787	71.53092141	131.9408407	168.0253084	113.6776796
2488-2L	39.17782437	5.669587103	28.54772038	40.97238106	59.24867777	39.76975321	117.511072	161.4027406	64.29078914	29.73761636	71.02845289	131.935515	168.4273103	112.4029195
2488-2R	39.0800037	5.66157331	28.56761645	40.81606322	59.51457181	40.88087224	118.5889261	161.682781	64.06343148	29.43813568	72.9971834	132.4010135	169.4666443	113.6994459
**2492-mid**	38.53495555	7.438023112	26.28833816	41.60662985	57.12186613	42.98845352	119.3204686	149.1876093	60.49253699	32.79309683	70.23417287	125.6175512	160.7187529	114.4101224
2492-1L	39.15965081	8.442331223	26.33233629	41.14289184	57.08407886	42.11669784	119.2888464	149.0547435	61.37732128	33.43819695	66.19434483	126.3045818	159.7718334	114.0587203
2492-1R	39.09959444	7.889360665	26.07513941	42.22223454	57.21030311	42.59511039	119.6571082	149.2876642	60.10541221	33.27377929	67.67420486	126.2637184	161.0249569	114.404584
2492-2L	37.75331234	7.789730535	26.62221472	42.01288705	57.45216773	41.94484229	120.1191742	147.9061752	63.67856872	31.77059297	64.48384976	126.3053294	160.8381296	114.4768419
2492-2R	39.36888582	8.533568628	26.02237547	40.67532584	56.67575446	43.15383436	117.568254	152.325319	59.96999993	33.33397817	65.90067466	126.5831012	160.148235	113.820268
**2498-mid**	37.60497618	13.36871183	21.70038162	35.89633033	58.28254085	44.05938973	107.8061053	149.8194178	67.23470852	29.11092537	64.59228189	130.3130205	179.9687299	124.1510073
2498-1L	37.75678849	13.09312042	21.3724809	36.04580853	58.29494881	45.44107083	108.035286	149.3998103	65.20836801	29.6496472	68.23156925	130.5472215	179.2145769	126.1498333
2498-1R	38.15584085	13.18601758	21.34671671	35.77030269	58.00889704	44.342493	108.4903315	152.4161982	65.55543932	28.79341409	63.82852261	129.8091143	179.8382075	125.1374667
2498-2L	37.60425094	18.84478818	22.38890072	36.4490866	58.37816385	45.40114113	107.1536136	154.6093308	64.46735369	29.37188365	61.32859821	129.1592573	178.821054	126.3346315
2498-2R	37.1835123	13.95344487	21.98699385	35.52112685	57.74763182	44.67899287	108.5722412	151.0347344	67.25098954	28.46633051	65.27514408	130.0110522	179.3616958	126.1420067
**2563-mid**	36.74510337	9.785359819	28.23509769	40.49465477	64.50779244	39.35396801	123.9997549	140.0481801	65.12813183	30.12999519	66.63331166	131.8746491	176.6272708	124.1769144
2563-1L	34.36549692	10.60824025	27.07967314	38.88279105	63.86436792	39.81173306	126.8244412	136.4949833	65.29244149	29.58016003	66.96035181	133.133678	177.9582948	127.3412995
2563-1R	35.22145602	11.03668472	27.845515	39.02749888	63.88303075	38.89218311	125.3825732	137.7326017	64.15830123	29.25721519	70.44278577	131.9600385	179.0328423	126.8754416
2563-2L	35.94695338	10.92894905	27.37098842	39.02893196	64.04273449	39.91401757	124.686691	136.0306533	64.90730471	30.10661539	60.7442917	131.1337787	179.413839	126.1608602
2563-2R	36.25178424	9.42989009	27.21455453	38.9112944	63.52045167	39.14951012	127.070563	137.9541092	62.34498254	29.67135581	74.15024799	132.1256231	176.8899094	126.1433758
**2565-mid**	37.52510171	6.445625484	26.28556588	40.05404954	56.42715921	42.95138241	109.4431656	169.2832894	59.01634179	31.54006689	74.22605856	128.9111258	151.4654744	108.5836497
2565-1L	37.32324549	6.227198427	27.72413595	40.32231776	57.20221022	42.48645421	105.8825169	170.5492857	58.9618181	30.67465687	68.6898471	126.8599133	151.2500888	112.3948558
2565-1R	37.47753941	6.016774937	27.21397354	39.54487303	56.86093267	42.0048087	102.238479	169.4866377	59.24035186	31.42489983	76.1672241	128.2209354	152.7683307	109.7386042
2565-2L	34.69246138	6.023009118	28.27812593	42.62327347	57.8880233	44.86535921	105.8295265	165.7079293	59.67196245	30.62780299	75.19577303	129.8213007	158.2032318	109.4099268
2565-2R	37.1794739	6.384787887	27.45524073	39.99507012	56.03927134	42.8266446	102.6497497	166.8844675	60.22093119	30.99302886	74.54685812	126.5317083	156.678866	112.111102
**2588-mid**	37.71559381	4.113683591	27.78400023	36.26690712	58.01202596	41.87790589	113.1448109	145.6352744	63.33583496	27.88212158	68.23681122	122.6714699	169.7484619	127.6127027
2588-1L	37.18991759	3.960873673	27.3280776	36.54156607	57.81333514	41.42982846	118.7280016	142.2979395	64.57073077	26.53339841	70.52010582	129.0217545	163.7640536	127.2422823
2588-1R	37.98111077	4.013511458	27.53103295	36.97961155	57.90344115	41.21102806	109.9189417	152.4644391	63.41646232	28.9372364	63.87600933	124.0419614	167.3790369	126.5728029
2588-2L	35.85476175	3.43253659	27.87954337	36.88729216	58.17210502	40.04181088	119.4415299	143.8486606	62.18137665	23.68386213	76.8983095	123.0620257	163.7477511	125.4094784
2588-2R	39.03182729	4.414603712	26.49629859	34.41469297	55.88877942	38.77115943	110.3307929	151.1778697	63.08403103	29.7168869	63.94849685	122.5001004	167.3323511	130.634752
**2600-mid**	37.54788318	6.20005117	24.26410081	35.41192866	52.53770082	38.0019013	118.012527	138.4491784	55.1388575	30.60241704	71.44257256	125.3562048	165.1616657	118.9733948
2600-1L	37.55324103	6.456923414	24.08575976	35.3301596	52.72046879	36.77551544	114.0842518	138.4534696	57.24190596	29.96891478	68.33179332	125.2550721	165.0317157	119.0496955
2600-1R	37.21335534	6.455031401	24.48314883	35.84574842	52.76270447	38.25355291	119.2008555	138.2119966	54.19972851	29.16719387	75.25225638	125.3074781	162.2691499	119.2870909
2600-2L	37.0528711	6.432539767	24.67033838	35.37363376	52.49508229	37.42509179	117.6036935	137.9233028	53.79983345	28.21327736	70.60976868	125.1646661	165.4669248	119.4388285
2600-2R	39.03729188	6.363466835	24.32250269	36.58180092	52.43714196	38.60588751	117.658907	135.8544534	56.40213361	30.87278022	58.99771235	126.673632	164.7899366	118.6600322
**2668-mid**	38.30658767	9.79170094	25.79045969	39.65785943	60.86540699	45.81172588	120.5189269	163.4314181	69.04848123	28.06755273	63.83605863	125.6628926	160.8786527	121.9621963
2668-1L	39.13785604	9.317858914	26.53175065	39.3729156	60.91072487	45.85128098	119.4000088	162.575049	69.23777333	28.85470432	62.57380852	127.5940563	164.6748123	122.9073005
2668-1R	37.74419496	9.300353682	26.2091216	39.02619657	61.67196607	45.71682037	119.3976642	163.5045031	70.00250728	27.39306335	63.58144538	127.730704	159.914846	124.2223657
2668-2L	35.93100721	9.09735325	26.94936353	39.48318895	61.34030885	45.82389564	119.9998111	161.4519346	70.11110653	25.27470485	61.66792946	127.834737	162.470947	123.1480396
2668-2R	36.49385397	8.92163007	26.61646262	41.31034662	60.77389288	45.96890659	118.4561828	163.1670523	66.82438169	25.55148243	67.63428796	128.5781613	162.3900554	122.5485323
**2670-mid**	32.07743452	-0.931879807	28.1066974	32.85627835	57.41582035	37.12768763	115.8905078	146.8752876	60.19779584	22.30217608	67.8109993	123.9673421	159.5836704	128.6591496
2670-1L	31.11048484	-0.326855923	26.9036237	32.14740708	56.36292259	37.42399894	117.4815207	145.7474438	62.1138962	21.522067	64.28582969	125.370641	160.3294729	128.626796
2670-1R	31.25806528	0.08974144	26.83269053	32.46957472	57.08957787	36.50865106	116.6307741	147.9441722	58.43009063	22.13769376	64.32442292	123.3327826	161.6374209	126.705293
2670-2L	31.69679108	0.299854092	27.79508952	33.27568576	57.28688329	37.95837024	119.1266831	146.5147127	61.08536724	22.20486796	66.11377143	125.5905643	162.0690935	128.5895929
2670-2R	32.40285402	-1.423798162	28.14479269	33.27401346	58.05253218	39.05232489	118.5336254	147.3924961	60.25942205	22.58829663	67.84089863	124.0540237	162.4901876	128.0891325
**2678-mid**	34.79447402	8.067628875	23.43783487	36.77080102	55.35896339	38.94821633	112.5869006	149.8524937	53.06903525	30.90540583	61.92365409	122.0778596	159.559099	119.7705567
2678-1L	35.8445514	8.135080234	23.62608919	37.8437054	55.60981927	38.23187988	113.6883918	152.9806473	53.57897478	30.89532321	62.63157783	120.6297068	162.2321633	117.3095227
2678-1R	34.20537998	8.380069871	23.27852347	38.02307769	55.83072617	39.24743163	113.1238337	148.4384761	53.25911705	30.76718538	59.28753914	123.0847585	160.9863675	118.4941078
2678-2L	35.37250526	7.584465945	24.2158774	37.45294304	55.19735152	38.37266445	112.5564581	150.3876568	53.99369377	30.91902429	64.50837241	121.5761292	161.2863225	118.3222373
2678-2R	34.16325994	8.107449037	24.37934167	38.39587088	55.92019993	38.87201965	111.7423757	150.3136207	54.40747637	29.82645709	62.90320693	123.5634878	163.2234479	116.969351
**2683-mid**	37.2847452	13.10396361	24.50873716	37.0566022	55.31992471	40.39204343	121.3214158	147.0942305	61.42750296	24.79423401	76.34575062	126.0466314	159.388519	123.249699
2683-1L	36.99989992	11.96001767	25.08593864	36.53244652	55.70338067	40.59768252	119.0407444	147.6428857	62.48518807	25.0686323	78.88611689	127.2532514	157.5028329	125.2078064
2683-1R	37.35241375	12.47768934	24.77983704	36.44623935	55.41896926	40.53984573	120.0696243	146.558094	62.08386789	24.43787822	74.9750166	126.3045801	161.5834417	124.9826817
2683-2L	36.71116402	12.33123073	25.16689314	37.3411189	55.74014607	41.99009113	119.156992	146.2853535	61.95140247	24.40706312	75.33684972	126.9395299	157.4944765	122.6859154
2683-2R	38.0813607	12.34132464	24.484529	37.07626564	54.88351799	41.0567404	121.720571	144.2874336	61.29980765	26.07377629	78.26213035	127.4359512	160.9970786	123.5421437
**2695-mid**	33.69420907	8.575916592	26.21535233	34.20422177	55.85465746	39.45148197	129.2947826	145.2114272	59.35558272	23.23340665	67.76652222	124.763224	170.8644039	137.2537672
2695-1L	33.31296822	8.831962063	26.45748575	34.02491898	55.57285709	39.6319765	130.9686704	143.7618023	59.22205178	23.0077261	69.01224261	123.3350127	167.3238965	137.7144322
2695-1R	32.89857037	8.942159464	26.75420584	34.18379072	54.81891885	39.63855763	132.2706445	142.5054395	58.7436093	23.07105936	67.14010143	123.6457592	170.6772688	137.2737926
2695-2L	33.14366868	7.883734597	27.57108057	33.87758276	56.25234059	38.85956681	127.9586072	146.3168544	59.41753242	21.63179365	71.88937964	123.4001969	167.1476729	139.5328616
2695-2R	32.49734187	9.474130266	26.62416084	34.15282475	55.26009748	39.08759028	131.8295928	144.5277112	57.91719252	21.96649999	71.07229896	123.9004943	168.1697193	136.1189579
**2702-mid**	36.45126877	9.689309369	28.53859827	35.01417554	54.96637947	41.57534823	113.5734764	146.4001022	59.44922112	28.98453183	69.54893105	122.579609	162.8452926	126.2848409
2702-1L	36.86936878	5.70085003	28.33333785	35.09449598	54.79391991	41.13935008	111.7388595	143.6953762	57.93448353	28.44500116	70.25791324	124.0365752	161.2578745	126.0315342
2702-1R	36.9491976	9.047773731	29.49664527	35.48950291	55.30350674	41.74197959	110.4593511	148.1475357	60.49685218	29.32457225	72.25530438	122.5089315	161.9886844	126.8630475
2702-2L	36.58288599	4.779469868	28.68327041	35.40008546	55.14911968	42.0814802	113.5241312	144.2726018	58.12320482	29.63353774	65.39017087	124.1326385	160.3864174	127.9915618
2702-2R	36.62968297	8.980496143	28.65717768	35.01722632	55.22795475	41.91262564	111.3676506	146.9175498	60.42979399	29.23844441	65.26306578	123.2971938	163.5761629	127.9973598
**2703-mid**	33.35844845	6.833100707	25.97529706	34.81454983	50.16519413	40.77766927	108.4035013	155.4073845	52.33068682	22.25713103	75.98293354	121.4175916	156.5838969	118.6676602
2703-1L	32.59402752	6.723481023	26.08420977	34.86791709	50.63027892	41.60095451	108.537649	157.4925723	51.61361366	22.05984486	76.04481242	121.6189357	155.9695774	119.7574
2703-1R	31.61902357	5.183551593	26.36100435	34.88924698	50.62873905	41.76477175	110.3353596	154.5269859	51.79817301	22.45709192	70.71872453	121.333311	157.8968254	122.5423894
2703-2L	33.39480895	6.500618598	26.69147601	34.42117972	50.83966814	40.98196276	108.8335686	158.7575146	52.11346071	21.81628142	73.3783415	122.3063699	157.3725067	120.3434434
2703-2R	32.27077423	6.400510249	26.23516387	34.80468503	50.60151677	41.35089451	109.7894325	155.9273266	50.79836377	21.02662955	75.3330707	122.8055204	157.3356578	121.3905127
**2708-mid**	35.23814381	14.50103671	30.95003584	42.88145507	62.68380073	45.07050684	119.0014253	157.3436546	65.13871084	25.18378701	64.76978769	132.3581392	160.6126425	115.3568318
2708-1L	35.55198203	14.73802462	31.42420282	43.14704352	63.50166396	45.11720799	117.5928483	154.7026326	66.06204942	24.77017085	68.71188474	131.530658	163.126963	116.0527474
2708-1R	35.00338108	13.02059576	30.97792277	42.6142849	62.86939044	45.72483403	116.1912018	153.6230136	66.4672414	25.53387019	70.08594471	132.9104009	158.2254928	115.8033471
2708-2L	35.36211626	15.08177429	30.78317441	43.98593197	62.90286636	45.04428268	114.7164394	158.5770972	65.73533236	20.1081496	66.39858027	131.9125506	163.160604	115.1398634
2708-2R	36.07634166	12.43117883	31.01304741	43.35566942	63.19675642	44.45217654	116.939169	156.4526026	65.0086023	25.04850964	71.75694248	130.8005273	159.4028898	116.7381409
**2713-mid**	33.29602491	7.063010572	29.85713389	39.03572713	62.26159446	45.70222066	100.7747268	149.526074	69.3817834	28.81640953	75.57184203	131.5201201	172.358242	122.1970254
2713-1L	34.18542268	7.857983163	29.79402055	39.85120032	63.09257376	45.15478179	96.00110967	150.4904474	68.21304652	28.01890198	76.15894417	132.411927	171.443407	119.4918139
2713-1R	34.12982609	7.170114776	30.03532367	39.73671228	62.11740007	45.71552311	98.26063604	149.4520024	68.9246225	28.63490536	74.59298828	131.9263234	174.7479362	121.218759
2713-2L	32.92222333	7.255544698	30.52996983	39.79970063	62.79660164	45.03682026	97.20013867	149.4978548	69.97146044	26.41917017	74.24709112	132.2845567	173.2623839	120.0795894
2713-2R	33.2575141	6.473873875	27.27851613	40.0804111	61.89912472	45.50003086	97.73796042	150.506304	68.61109283	28.29452437	72.04273552	132.7365732	176.7554274	119.8412609
**2721-mid**	35.89181018	2.753869354	30.63096797	43.06439673	61.13472581	43.05087996	123.6074122	151.9981548	65.30144977	29.85218901	73.65336013	132.9715188	164.9755228	118.0643037
2721-1L	35.29610186	2.969937467	29.75218306	42.35745846	61.01143093	42.9008029	124.3149764	150.4638735	64.2286075	30.1445644	74.07507991	131.8283917	167.6328201	117.9038351
2721-1R	35.51217951	2.452085866	30.32499113	42.95501533	60.81534447	43.41562493	123.7007562	151.2919661	64.07908447	30.52576608	75.50949197	132.4925858	167.6588597	116.9036688
2721-2L	36.00911054	3.266262311	29.72831623	42.29941735	60.82697249	41.91823364	124.389991	151.410221	63.91251518	30.40030713	75.91664139	132.9775646	167.718529	117.1805587
2721-2R	36.36671443	3.862499214	29.12029372	42.6444096	59.85908139	42.78801472	123.3354301	152.3429714	63.6595759	30.6225232	66.3221981	133.1402498	167.7502048	114.0579311
**2724-mid**	34.09862892	19.04006359	21.23774285	34.48823025	51.33934596	36.67569742	117.6323905	155.1876021	55.23226324	25.81704554	65.20127264	121.417475	154.3318838	113.7995227
2724-1L	34.74329435	18.87259948	21.99086137	34.58436765	51.09260789	39.4898504	114.8158785	159.7351594	54.24104139	26.77251965	68.13178363	121.645436	153.41794	111.7794561
2724-1R	34.76016479	18.78739216	21.05737698	35.22752084	51.11485311	36.88422088	115.065068	159.7816851	53.54625318	26.92958711	70.75414713	120.1869866	152.5570727	111.9158989
2724-2L	34.99108156	18.26966703	21.48916974	34.97885826	52.65455385	40.33252995	113.0591901	156.0749499	53.18699746	26.14012855	69.50405075	122.549662	154.1498917	111.3134852
2724-2R	33.29667171	17.34396801	21.05903598	34.61028866	51.52348146	37.22398103	118.6431951	155.8879805	52.55935803	25.40166615	65.5363571	120.6077813	154.487785	115.324802
**2726-mid**	33.03887067	5.341899676	26.65056042	35.45963664	56.54133819	40.18111152	121.2165017	160.9213996	64.82525391	26.83560991	66.1666173	121.7766946	159.1955572	122.0387327
2726-1L	33.86451718	5.70258256	26.51290782	35.51067536	56.10127753	39.965652	118.5566564	159.5468734	62.93118707	27.10784907	63.06235124	122.5241012	158.9855366	121.4520834
2726-1R	34.25002088	5.113148832	26.72928573	35.40555427	56.98407789	39.10535509	120.7770266	161.5140741	62.38706403	27.08330543	63.09271433	122.1885083	161.3580871	120.7647892

2726-2L	33.33829835	6.023484262	27.04863572	35.4453345	56.95680507	40.38201623	118.5221508	159.0280133	64.60491135	26.12569466	61.64762823	119.721727	160.1423943	122.0400777
2726-2R	34.85114896	5.740089272	26.65452738	35.17769894	56.74663103	38.6819439	119.7530238	160.1479402	64.24707983	27.97347367	63.52939214	120.417555	160.4275714	122.2859208
**2767-mid**	32.61561482	1.949178963	30.71169043	42.05848711	57.92665173	41.38579909	120.0837853	146.7778446	66.72085135	28.89286709	64.01367572	128.7007635	162.6876576	119.8856283
2767-1L	31.34704105	2.239726472	30.27701412	42.3911521	58.39107473	41.3210615	121.7606846	146.2255085	65.81815353	28.86543257	63.80729134	129.1346521	161.0206453	119.9587651
2767-1R	33.34869856	1.370872701	30.57968931	41.2892767	57.68895226	39.33043884	121.9651679	144.3115261	63.79636304	29.35191073	60.66417586	128.3418758	164.8459526	121.8554648
2767-2L	30.82432083	3.116989783	29.68311562	42.59030456	58.21890631	41.41652342	123.0623987	138.384757	60.6416445	27.64077645	65.5339366	127.67727	162.1842137	125.9381684
2767-2R	33.78693628	0.772492701	30.2483496	41.2361213	57.67664029	38.67324517	123.0505449	144.1669334	61.31128714	28.38345617	64.68906149	127.7028247	168.7018229	123.5358869
**2780-mid**	34.23315269	2.166687426	29.60833086	38.72987149	61.22439309	41.1099501	122.2456588	147.4487886	63.9688418	28.00928955	75.38869134	129.683385	164.4033833	121.7944671
2780-1L	34.57446952	1.339856579	29.82313071	39.7808046	61.28107531	41.85066347	124.7106572	148.1580156	62.08613516	28.22064412	77.67441268	129.113044	165.8267816	121.3696645
2780-1R	34.63844245	1.838830827	29.51788477	39.45823036	60.98264573	41.37534635	128.1902866	145.2439458	63.71705322	27.87594219	79.33119591	129.4987859	165.9653885	119.5560628
2780-2L	34.40429986	6.270390254	29.50068989	39.27695776	61.17671649	41.62102166	122.2067282	147.1991408	62.4155283	28.17460084	80.56677759	130.4799169	165.4515362	119.8890604
2780-2R	35.61926019	1.932136746	30.35639771	40.89414562	61.5835018	41.94317436	127.5801584	147.6835319	61.77937372	28.84318347	75.22546729	130.9864923	164.6506732	115.547984
**2815-mid**	32.37513438	4.60407876	32.35529049	42.90175457	62.9161649	43.28169982	119.1168274	167.1514443	63.06765992	26.74686466	69.60302148	134.3109962	158.9181042	110.4447814
2815-1L	31.53371074	4.883662034	31.94903317	42.73373183	62.09795127	43.89705625	117.9697419	168.0243723	62.28800239	25.71880337	68.56391134	134.1146585	157.6720625	107.9211968
2815-1R	32.78341246	4.554630321	32.03903796	42.52513293	62.3941674	42.98988041	118.1731612	167.2899534	62.71623698	26.13955165	67.87243279	134.3965182	158.817603	109.9199706
2815-2L	31.57399268	5.204860531	32.00924663	42.31856921	62.97745384	41.96046156	118.2934223	168.0210216	62.89038773	26.15995652	69.23503101	134.4680706	157.7455394	106.8670808
2815-2R	32.24840335	4.86744784	31.9444481	42.69738569	61.97980738	43.43535322	118.8540438	166.2543329	62.63976502	26.29918535	72.59400318	134.5772298	159.3218793	108.994114
**2823-mid**	38.3892745	7.27643971	24.46940575	37.66076617	52.6286348	37.75558915	118.3173693	149.4932298	59.3926301	30.50333699	76.97152236	119.1129132	159.6265514	108.2471512
2823-1L	38.41503849	5.816299123	24.7803932	37.83121089	52.71103953	37.90863315	120.1301836	147.8110506	58.97065174	29.96641206	73.81471082	119.2893975	158.8269113	109.3199193
2823-1R	38.6577566	7.654521564	25.15421348	37.90215713	52.97047288	37.45712617	121.8859859	148.933087	57.41541589	29.58738894	70.29713686	119.5038746	160.2447763	108.0079494
2823-2L	38.25261666	3.806104115	24.20649258	38.93446068	53.22640567	39.41799469	117.9060204	153.9286297	59.68023438	30.52060714	74.79381364	120.0704655	157.8646174	106.9818526
2823-2R	38.00370825	3.855973311	24.42171306	38.9839802	53.06545144	40.11207105	117.3252651	153.3122856	58.01100327	30.52951799	71.88829019	119.7249751	156.9768629	105.4298189
**2834-mid**	32.61906087	9.058735204	21.33147171	35.85771668	53.45335784	36.9742762	119.8054305	160.8318292	60.01147046	24.27530951	64.01847827	121.4109831	158.4653005	121.6036584
2834-1L	32.34873404	8.858616539	21.47205314	35.92396544	54.02146088	36.85866966	120.1940188	162.3820905	60.55987104	23.97833399	68.2481865	121.4056352	158.0030271	121.4190436
2834-1R	31.87754163	9.908896005	21.12463865	35.45091968	53.26169798	37.06032568	120.2277032	160.782119	59.17507322	24.05621549	66.5603427	120.2085176	158.6663945	121.5278795
2834-2L	30.97750897	8.538666781	22.14462054	36.03833867	53.88775588	36.94554333	120.2880711	161.8882259	61.02825712	22.79750438	61.15887498	121.8602434	156.0519626	123.7064991
2834-2R	31.75110441	9.623509774	21.63964858	35.75733671	53.52991476	37.14921269	120.0651978	162.7126818	61.38412286	23.92967321	66.4083681	120.5623452	156.3889961	121.5008219
